# Host sequence motifs shared by HIV predict response to antiretroviral therapy

**DOI:** 10.1186/1755-8794-2-47

**Published:** 2009-07-23

**Authors:** William Dampier, Perry Evans, Lyle Ungar, Aydin Tozeren

**Affiliations:** 1 Center for Integrated Bioinformatics, Drexel University, Bossone Research Center 711, 3120 Market Street, Philadelphia, PA 19104, USA; 2 Genomics and Computational Biology and Department of Computer and Information Science, University of Pennsylvania, Levine Hall, 3330 Walnut Street, Philadelphia, PA 19104, USA

## Abstract

**Background:**

The HIV viral genome mutates at a high rate and poses a significant long term health risk even in the presence of combination antiretroviral therapy. Current methods for predicting a patient's response to therapy rely on site-directed mutagenesis experiments and *in vitro *resistance assays. In this bioinformatics study we treat response to antiretroviral therapy as a two-body problem: response to therapy is considered to be a function of both the host and pathogen proteomes. We set out to identify potential responders based on the presence or absence of host protein and DNA motifs on the HIV proteome.

**Results:**

An alignment of thousands of HIV-1 sequences attested to extensive variation in nucleotide sequence but also showed conservation of eukaryotic short linear motifs on the protein coding regions. The reduction in viral load of patients in the Stanford HIV Drug Resistance Database exhibited a bimodal distribution after 24 weeks of antiretroviral therapy, with 2,000 copies/ml cutoff. Similarly, patients allocated into responder/non-responder categories based on consistent viral load reduction during a 24 week period showed clear separation. In both cases of phenotype identification, a set of features composed of short linear motifs in the reverse transcriptase region of HIV sequence accurately predicted a patient's response to therapy. Motifs that overlap resistance sites were highly predictive of responder identification in single drug regimens but these features lost importance in defining responders in multi-drug therapies.

**Conclusion:**

HIV sequence mutates in a way that preferentially preserves peptide sequence motifs that are also found in the human proteome. The presence and absence of such motifs at specific regions of the HIV sequence is highly predictive of response to therapy. Some of these predictive motifs overlap with known HIV-1 resistance sites. These motifs are well established in bioinformatics databases and hence do not require identification via *in vitro *mutation experiments.

## Background

Human Immunodeficiency Virus (HIV) is a single stranded RNA virus that contains nine genes coding for fifteen proteins [1,2]. HIV has a powerful effect on the human immune system due to its ability to hijack hundreds of human proteins in continued infection [3]. HIV's POL gene codes for three important enzymes that are essential to the life cycle of the virus: the protein reverse transcriptase (RT) is common to all retroviruses and transcribes the viral RNA into double stranded DNA [1]. The RT enzyme has no proofreading ability [4] which explains the high mutation rate observed with *in vitro *experiments for the HIV virus [5]. POL also encodes the integrase protein which fuses the viral DNA produced by RT into the host genome [4]. The third enzyme coded by POL, protease (PR), is an enzyme that cleaves the multiple proteins coded by HIV's GAG and POL genes into separate functional units [1]. Mutations at the active sites of these three enzymes or inhibition of enzyme activity by drugs disrupt HIV's ability to replicate in host cells and thus block the infection cycle [6].

Most of the drugs that are currently used for controlling HIV infection target the three viral enzymes coded by the HIV POL gene. Antiretroviral drugs such as zidovudine (AZT), lamivudine (3TC), emtricitabine (FTC), zalcitabine (ddC), stavudine (D4T), didanosine (DDI) and nevirapine (NVP) target RT [7] whereas antiretroviral drugs such as indinavir (IDV), nelfinavir (NFV), and atazanavir (ATV) were designed as PR inhibitors [8]. Clinicians also use a set of entry and integrase inhibitors in HIV treatment [9-11]. When antiretroviral drug are used one at a time, eventually a drug resistant viral phenotype will emerge [12]. Viral loads (VL) from *in vitro *cultures of HIV infected immune cells have diminishing growth rates in the presence of antiretroviral therapy but eventually a resistant viral phenotype emerges [13]. The resistance conferring mutations in the viral genome have been extensively documented and these mutations have been correlated to response to therapy [13-16]. Combination of antiretroviral drugs has the advantage of targeting multiple stages of the viral life cycle. The multi-target Highly Active Antiretroviral therapies (HAART) exert a high level of evolutionary pressure on the virus by effectively requiring multiple simultaneous mutations to produce resistant strains [17-19]. As a result, the virus takes much longer time to develop resistance to several drugs at the same time [20].

HAART therapies often reduce viral replication to undetectable levels. They decrease morbidity and mortality rates but nonetheless can be ineffective in some individuals [21,22]. Search for new antiretroviral drugs with different target sites along the HIV sequence is ongoing. Targeting the virus itself may not be enough, however, to block the progress of infection. One may also have to consider the set of host proteins playing crucial roles in viral replication as targets for therapy. Recently, researchers have identified sets of human proteins that interact with HIV proteins [23-25] and another set of host proteins required for HIV infection through a functional genomic screen (([26-28], but the modes of interaction of these host proteins with specific HIV proteins are yet to be fully explored. Nevertheless, the ability of HIV-1 viral proteins to bind within the host cell network is likely to play a critical role in disease progression [29]. It is possible that this new focus on host proteins interacting with HIV will lead to new therapies targeting host cells required for HIV infection [30].

In this study, we first cluster patients into responder and non-responder categories based on viral load response to antiretroviral therapy. We then used stepwise logistic regression to differentiate responders and non-responders using linear sequence motifs common to host and viral genomes as features. We focused on viral load in the responder/non-responder classification because recent studies indicate that CD4 cell count monitoring does not accurately identify individuals with virologic failure among patients taking antiviral therapy [31]. A novel aspect of our study is the recognition of bimodality [32] in the viral load reduction in antiretroviral therapy in patient data stored in the Stanford HIV Drug Resistance Database [33] both at eight weeks and twenty four weeks after the beginning of the therapy. In total, we used three different methods for assigning responder phenotype based on viral load. Multiple models of phenotype classification allowed us to identify the role of phenotype selection in determining significant features associated with drug response.

Another novel feature of our study is the treatment of drug response as a two body problem, namely that response to drugs is assumed to be affected by both the viral and host genotypes. We sought to identify linear motifs on the HIV sequence that are also found in the host and are functionally annotated: host transcription factor binding sequence motifs [34], miRNA binding sequence motifs on the nucleotide sequence [35] and eukaryotic linear motifs [36] on the protein amino acid sequences. The motivation to use such features in predicting responder/non-responder categories comes from the observed phenomena of the virus hijacking host cell apparatus for its self replication [37]. Another important motivation is to find a feature set based solely on viral sequence and not requiring *a priori *information obtained via virus-specific *in vitro *cell assays. This type of a feature set is attractive, as it can be used to explore the drug response of viruses to antiviral therapy in the absence of extensive data on resistance mutations. Previous research on quantitative prediction of patient response to antiretroviral drugs in HIV infection [38-43] has employed similar and even more advanced machine learning algorithms than used here, but has not made explicit use of biologically meaningful linear motifs.

## Results

### Responder/Non-responder classification

**Figure 1:**
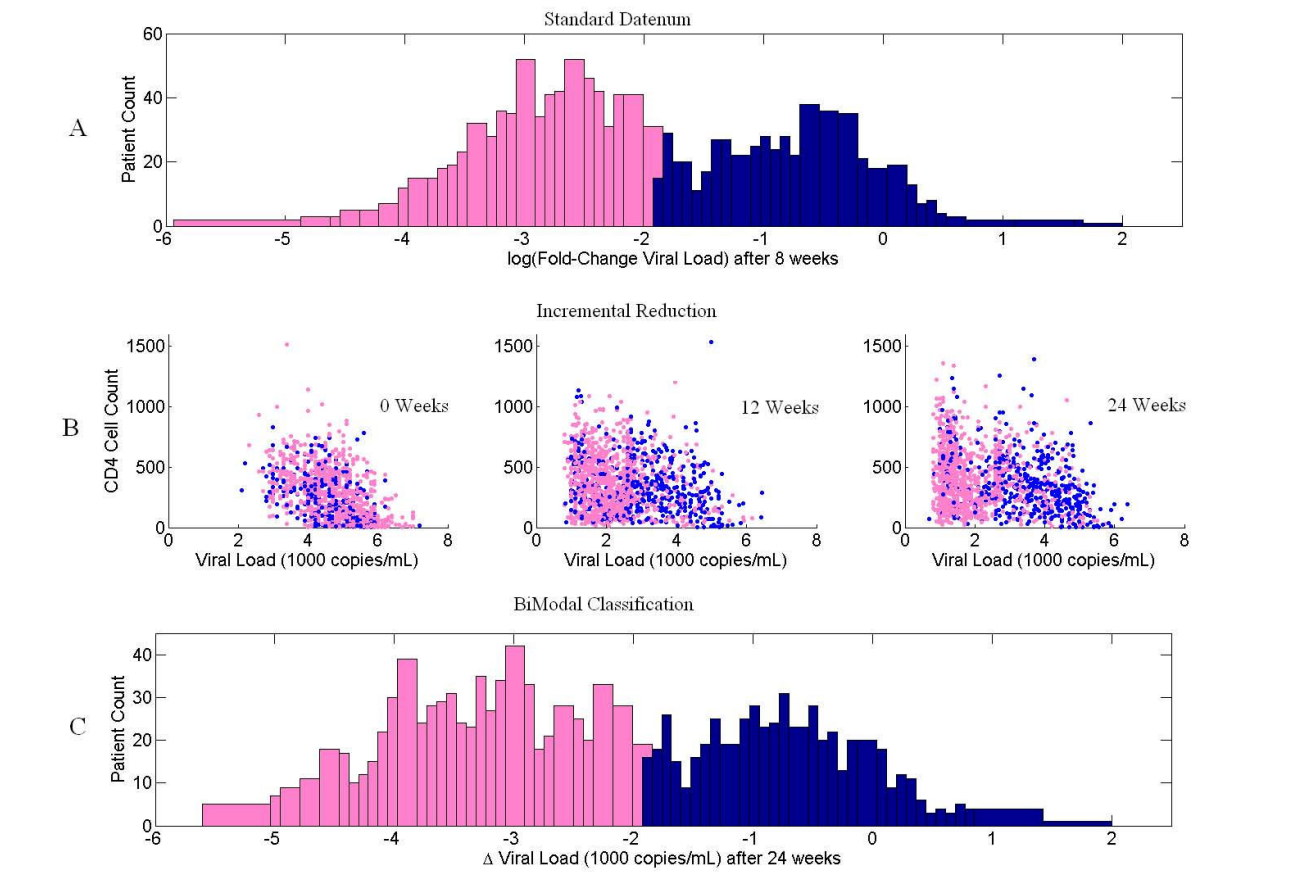
**Responder Classifications**. A graphical representation of the three phenotype classification methods: Standard Datenum (SD), Incremental Reduction (IR) and Bimodal classification (BM). Figure 1A: SD, A histogram showing the log10 change in viral load of all patients in the database. Patients labelled as "responders" are marked in pink and non-responders in "blue". Figure 1B: IR, Three scatter plots representing the viral load vs. CD4 counts for all patients in the database after 8, 12, and 24 weeks of therapy. Patients which decreased in viral load in 75% of their visits are labelled as "responders" and marked in pink; those that did not are labelled as "non-responders" and marked in blue. Figure 1C: BM, A histogram of the change in viral load after 24 weeks of therapy. Those patients that decreased by more than 2000 copies/ml were labelled as "responders" and are marked in pink; those that did not were labelled as "non-responders" and are marked in blue.

Clinical annotation of more than 2,000 RT sequence samples in the Stanford HIV Drug Resistance database contained measurements of VL at six time points during the course of twenty-four week therapy. The drugs used in various single and combination therapies as well as the numbers of HIV-1 individuals taking the therapy are shown in Table 1. As described in the methods section, the first classification method for responders and non-responders, SD or Standard Datenum [39], was based on the fold-change of the entire patient database between the 0 and 8 week time points. The SD method classifies patients as responders if their viral load decreases by 100-fold over this time period. All other patients are labelled as non-responders. As shown in Figure 1A, this led to binomial distribution with clear peaks identified for responders and non-responders. The second method for phenotype classification, Incremental Reduction (IR), is based on patients having a reduction of viral load in four out of six weeks. Figure 1B shows the sub populations of responders and non-responders for this classification as a function of VL at three different instances in the clinical trial. It is clear from the figure that responders move towards zero VL whereas non-responders are much less mobile in this setting. The third method for phenotype classification (BM) was based on the observation that viral load reduction after 24 weeks of therapy exhibited a bimodal distribution (Figure 1C). This method used a cutoff of 2,000 copies/mL to differentiate between responders and non-responders. Subpopulations corresponding to each drug regimen shown in Table 1 also exhibited similar bimodal distributions.

**Table 1:** Therapy Classification

	**Standard Datenum**			**Incremental Reduction**			**Bimodal Classification**		
	
	R	NR	Mean AUC	R	NR	Mean AUC	R	NR	Mean AUC
AZT	526	390	0.7750	581	335	0.8550	395	521	0.7802

AZT, IDV	182	148	0.7803	189	141	0.9281	144	186	0.9107

DDI	466	273	0.7572	503	236	0.8363	272	467	0.7648

DDI, NFV	249	130	0.7352	264	115	0.8004	175	204	0.6814

D4T	450	307	0.7654	482	275	0.8081	274	483	0.7683

D4T, NFV	266	153	0.7499	280	139	0.6664	181	238	0.6713

D4T, NFV	372	200	0.7377	391	181	0.8455	260	312	0.7613

D4T, DDI, NFV	234	115	0.7518	242	107	0.7764	173	176	0.6817

3TC	582	466	0.7721	654	394	0.9280	408	640	0.7788

3TC, IDV	187	151	0.7748	196	142	0.9030	144	194	0.8763

3TC, NFV	202	159	0.7535	242	119	0.8810	175	186	0.8606

3TC, AZT	509	379	0.7731	560	328	0.8439	391	497	0.7845

3TC, AZT, IDV	177	145	0.7849	184	138	0.8858	144	178	0.8815

DDI, EFV	248	121	0.7389	208	89	0.9312	192	177	0.6711

D4T, EFV	260	125	0.7406	285	100	0.8479	194	191	0.9887

D4T, DDI, EFV	233	107	0.7516	254	86	0.9446	188	152	0.7499

3TC, EFV	207	130	0.7313	245	100	0.9731	179	166	0.9497

All Therapies	1115	904	0.7644	1188	831	0.8351	700	1319	0.8402

The overall statistics of the clinically annotated reverse transcriptase sequences from the Stanford HIV-1 Drug Resistance Database. The table shows breakdown of patients in each therapy regimen using the three different classification rules: Standard Datenum (SD), Incremental Reduction (IR), and Bimodal Classification (BM). R; responders, NR; non responders. The average AUC over 500 training/testing iterations indicate the success in differentiating responders from non responders using short linear sequence motifs as features in machine learning.

**Figure 2:**
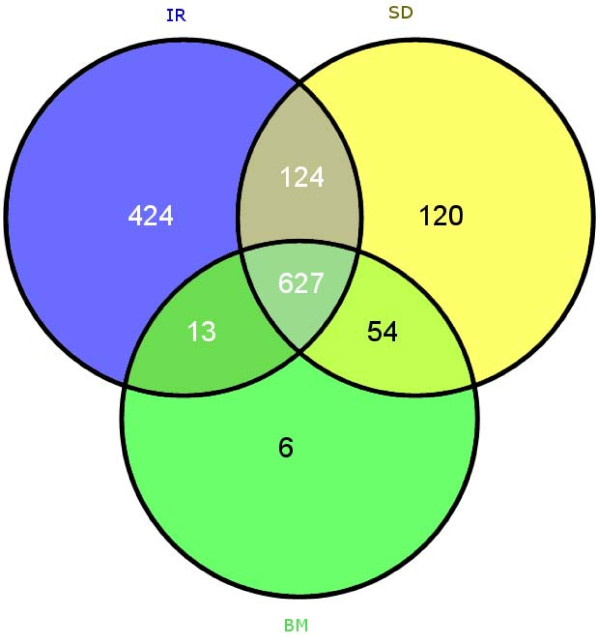
**Venn Diagram**. Venn diagram showing the intersection between responder sets corresponding to SD, IR, and BM classification.

The overlap between these three methods is shown in the Venn diagram in Figure 2. More than half of the responders from each method are also declared responders by the other two methods. However, 244 of the 925 patients labelled as responders by the SD method at eight weeks are not considered responders after 24 weeks by the BM. This suggests that after a strong initial response to therapy, some patients regress between 8th and 24th week of intervention with antiretroviral drugs. We used these three clinically relevant phenotype classification methods to identify sequence motifs associated with the responder group in each classification.

### Conserved linear motifs along HIV and their correlation with response to antiretroviral drugs

**Figure 3:**
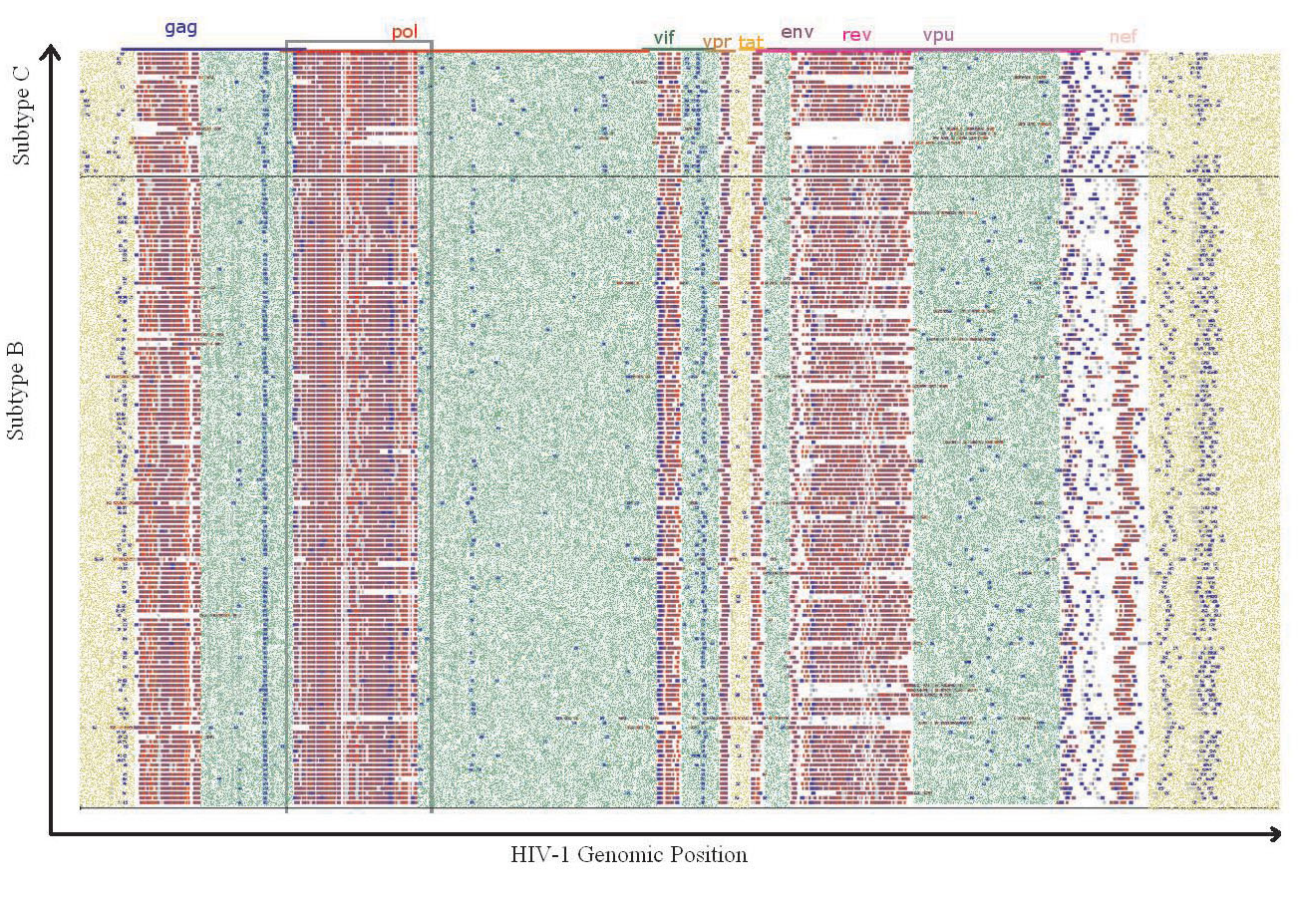
**Feature Annotation**. Annotation of a short linear motifs (Eukareotytic Linear Motifs, miRNAs binding sites, human transcription factor binding sites) along the viral sequence for 100 subtype C and 500 subtype B sequences. The colour code is as follows: homology Islands (green), human miRNA binding-sites (blue), human TF sites (silver), cleavage ELMs (red), ligation ELMs (purple), modification ELMs (brown), and export ELMs (pink). The clinically annotated sequence region is shown in the black box.

Our results show that the HIV sequence, although highly variant in nucleotide sequence, expresses eukaryotic linear motifs (ELMs) that are largely conserved over hundreds of subtype B and subtype C sequences, as shown in Figure 3. The motifs recognized in globular domain regions are not shown as they are less likely to be instrumental in the interactions of HIV-1 proteins with host targets. The figure illustrates the presence of ELMs at high density along the flexible, domain-free regions of the HIV proteins. ELMs found on HIV proteins are largely conserved in frequency of appearance in eukaryotic proteomes (unpublished observations) and as such these motifs are good candidates in feature selection for predicting response to antiviral drugs.

We used Step-Wise Logistic Regression (SWLR) to classify patients into responder or non-responder categories based on the presence or absence of ELMs, miRNA binding sites, TF binding sites, and resistance sites, collectively referred to as *features*. SWLR employs an iterative algorithm to determine which features should be included in the final logistic regression model [44]. In brief, the algorithm starts with an initial group of features and fits a logistic regression model. It then discards any features with a near zero coefficient and determines which of the excluded features may have a non-zero coefficient if added to the model. This process repeats until it converges to a solution; In our experience this occurs within 100 iterations.

**Figure 4:**
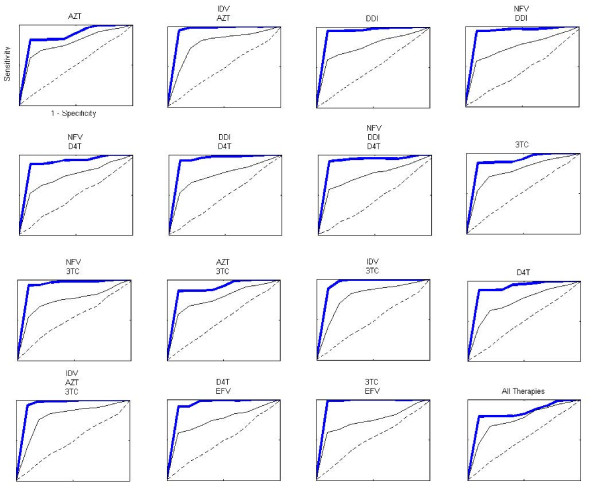
**ROC Curves**. Receiver Operator Characteristic (ROC) curves determined by the stepwise-logistic regression (SWLR) for the therapy regimens presented in Table 1 using the IR classification. The BOLD blue shows the average ROC curve over 500 iterations. The solid black line indicates the prediction ability with 20% shuffling of the responder v non-responder categories. The dashed line indicates the corresponding averages of completely shuffled responder vs. non-responder categories.

We used SWLR in 500 iterations of training and testing at equal proportions for all responder/non-responder samples shown in Table 1. The resulting Receiver Operator Characteristics (ROC) curves for IR classification for the therapy regimens presented in Table 1 are shown in Figure 4. These ROC curves show high prediction accuracy of responders with the features used in the model. The area under the ROC curve (AUC) is an indicator of the combined sensitivity (ability to detect true positives) and specificity (ability to detect true negatives) of the model. As shown in Figure 4, random mixing of the responder and non-responder populations by 20% drastically reduced AUC for all drug regimens. Random mixing by 50% resulted in AUC values nearly equal to 0.5 as would be expected for randomly selected populations. These results confirm the utility of the selected features for predicting responder/non-responder identity using logistic regression.

The AUC values for all three phenotype classification methods are shown in Table 1. Note that AUC values for BM and IR phenotype classifications are similar and point to high accuracy of prediction of outcome with these classification methods. The SD method, on the other hand, gave AUC values that were somewhat smaller than the other two methods. It is possible that the feature set used in our SD analysis is not optimal for predicting responders after eight weeks of therapy.

### Regression Coefficients

**Figure 5:**
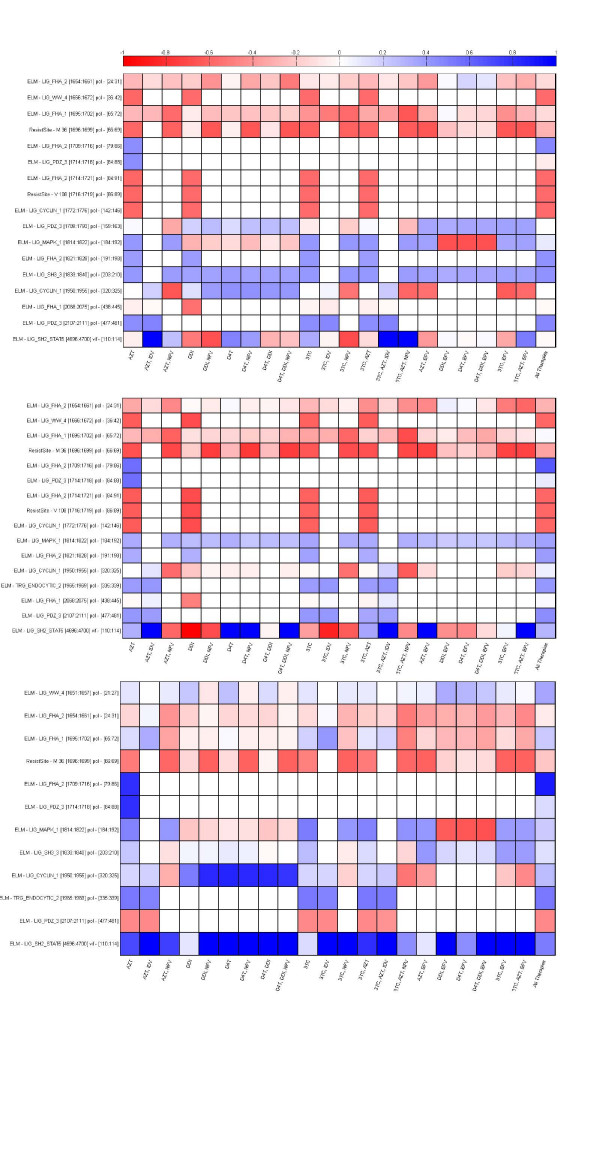
**SWLR Feature Regression Coefficients**. Heatmaps indicating the average of the SWLR regression coefficient for the motifs used in the classification. Blue colour in the ruler bar indicates that presence of an ELM motif creates greater likelihood of being in the responder category (R ELM) whereas red indicates greater likelihood of being in the non-responder category (NR ELM). Top Panel: SD; Middle Panel: IR, Bottom Panel: BM.

The average number of regression coefficients (features) found significant over 500 training/testing iterations ranged from five to ten, depending on the drug regimens presented in Table 1. These features corresponeded to two specific resistance sites (RS)s and ELMs. In a set of control SWLR computations, we used other motifs such as human transcription binding site motifs and miRNA binding motifs on the RT sequence, but none of them were found to be significant in regression. Shown in Figure 5 are regression coefficients with absolute values greater than 0.5 for the three phenotype classifications: SD (Figure 5A), IR (Figure 5B), and BM (Figure 5C). Note that the two resistance sites on the figure are highly predictive of outcome in single drug regimens such as AZT and DDI targeting RT along with the ELMs that overlap this part of the sequence. Mutation RS V108 is a strong indicator of poor response to AZT, DDI, 3TC, and AZT, 3TC combination at 8 weeks (SD classification) whereas RS M36 has a negative effect on a larger spectrum of drug combinations (Figure 5A). These two resistance sites are the only ones that emerged in the set of features that are highly correlated with response to antiretroviral drugs. However, the regression does not lose accuracy when resistance sites are excluded from the features used in the analysis (data not shown). In this restricted set the significance of ELMs overlapping the resistance sites increases to compensate for the deletion, confirming the important role this sequence region plays in signalling resistance to some of the antiretrovirals targeting RT. Our findings point to resistance sites (or overlapping ELMs) having strong correlation to response to single antiretroviral therapies, but response to HAART therapies are correlated strongly with functional host protein motifs that are also expressed by the RT.

One of the most consistent predictors of positive outcome across therapy regimens is the presence of ELM-Lig-SH3-3 (Figure 5A). This is the motif recognized by the SH3 domains of host proteins with a non-canonical class II recognition capacity [45]. The SH3 domain is a protein-protein interaction module commonly found in intracellular signalling and adaptor proteins. The SH3 domains of multiple endocytic proteins have been recently implicated in binding ubiquitin, which serves as a signal for diverse cellular processes including protein destruction [45].

The two resistance sites and the ELMs that overlap them continue to be predictors of negative outcome in terms of response to subsets of antiretroviral therapies in phenotype classification based on incremental reduction of the VL (Figure 5B, IR Classification). In this case, the consistent positive predictor is the motif ELM-Lig-MAPK-1. MAPK interacting molecules that carry this docking motif help to regulate specific interaction in the MAPK cascade [46,47]. It is feasible that human MAPK is recognizing the ELM on these RT proteins, decreasing their efficacy through phosphorylation or other inhibition methods.

Figure 5C, showing the BM classification method, reveals the resistance site M36 as a consistent indicator for negative response and ELM-Lig-SH2-STAT 5 as a strong indicator for positive response to antiretroviral therapy. This ELM is a motif recognized by proteins that have a significant impact on innate immunity during sepsis [48]. The innate immune system provides immediate defence against infection and serves as the first line of host defence during infection [49]. Recent research point to the depletion of white blood cells associated with innate immunity and their recovery under HAART [50].

Among the host proteins that have been documented to interact with the HIV RT protein, those that have at least one of the ELMs shown in Figure 5 are presented in Table 2. The table contains 33 host proteins with varying functions closely related to the immune response and signalling. The most common gene ontology categories [51] and KEGG pathways [52] among these proteins include adenyl ribonucleotide binding, phosphorylation, cell death, and apoptosis and pathways such as natural killer cell mediated cytotoxicity and the MAPK signalling pathway (Table 3). Our present knowledge of the grammar of protein interactions between the host and the virus does not allow us to draw definitive models of the network of interactions that differentiates responders from non-responders in HAART therapies. Nonetheless, the results presented above provide a start towards constructing a plausible mechanism of how viral and host genotypes affect response to antiretroviral therapies

**Table 2:** Interacting Proteins

**Entrez ID**	**Symbol**	**Gene Name**	**Significant ELMs Present**
59	ACTA2	actin, alpha 2, smooth muscle, aorta	The localization of the HIV-1 reverse transcription complex to actin microfilaments is mediated by the interaction of a reverse transcription complex component (HIV-1 Matrix) with actin, but not vimentin (intermediate filaments) or tubulin (microtubules)

60	ACTB	Actin, beta	Eukaryotic beta-actin binds to either the large subunit (p66) of HIV-1 reverse transcriptase or to the HIV-1 Pol precursor polyprotein in vitro; this interaction is believed to be important for the secretion of HIV-1 virions

70	ACTC1	actin, alpha, cardiac muscle 1	The localization of the HIV-1 reverse transcription complex to actin microfilaments is mediated by the interaction of a reverse transcription complex component (HIV-1 Matrix) with actin, but not vimentin (intermediate filaments) or tubulin (microtubules)

1457	CSNK2A1	casein kinase 2, alpha 1	Casein kinase II phosphorylates HIV-1 RT p66 and p51 in human cells

3439, 3440, 3449	IFNA1, IFNA2, IFNA16		IFN-alpha interferes with the initiation of HIV-1 reverse transcription resulting in a significant reduction in the relative levels of HIV-1 proviral DNA

3458	IFNG	Interferon, gamma	Up-regulation of LMP7 by IFN-gamma enhances proteasomal degradation of HIV-1 RT and presentation of the VIYQYMDDL epitope derived from HIV-1 RT

4772, 4773	NFACT1, NFACT2	nuclear factor of activated T-cells	NFATc facilitates HIV-1 RT reverse transcription activity and enhances HIV-1 infectivity in human T cells

5286	PIK3C2A	phosphoinositide-3-kinase, class 2, alpha polypeptide	HIV-1 RT heterodimer expressed in bacteria can be phosphorylated in vitro by several purified mammalian protein kinases including auto-activated protein kinase (PK), CKII, cytosolic protamine kinase (CPK), myelin basic protein kinase 1 (MBPK1), and PRKC

5578, 5579, 5580, 5581, 5584, 5588, 5590	PRKCA, PRKCB1, PRKCD, PRKCE, PRKCI, PRKCQ, PRKCZ		HIV-1 RT heterodimer expressed in bacteria can be phosphorylated in vitro by several purified mammalian protein kinases including auto-activated protein kinase (PK), CKII, cytosolic protamine kinase (CPK), myelin basic protein kinase 1 (MBPK1), and PRKC

5594, 5604, 6300	MAPK1, MAP2K1, MAPK12	mitogen-activated protein kinase 1	MEK1 in HIV-1 producer cells is able to activate virion-associated MAPK in trans, and the activated MAPK facilitates efficient disengagement of the HIV-1 reverse transcription complex from the cell membrane and subsequent nuclear translocation

5696	PSMB8	proteasome subunit, beta type, 8	Up-regulation of LMP7 by IFN-gamma enhances proteasomal degradation of HIV-1 RT and presentation of the VIYQYMDDL epitope derived from HIV-1 RT

6117, 6118, 6119	RPA1, RPA2, RPA3		Replication protein A and HIV-1 nucleocapsid protein interfere with the strand displacement DNA synthesis of HIV-1 reverse transcriptase by binding to the displaced strand and keeping it away from the newly synthesized strand

7150	TOP1	topoisomerase (DNA) I	Topoisomerase I (topo I) enhances HIV-1 reverse transcriptase activity in vitro and this effect can be inhibited by the topo I-specific inhibitor camptothecin

7157	TP53	tumor protein p53 (Li-Fraumeni syndrome)	Tumor suppressor protein p53 displays 3' -> 5' exonuclease activity, and interaction of p53 with HIV-1 reverse transcriptase (RT) can provide a proofreading function for HIV-1 RT

10527	IPO7	importin 7	Importin 7, an import receptor for ribosomal proteins and histone H1, is involved in the active nuclear import of the intracellular HIV-1 reverse transcription complex (RTC) containing HIV-1 RT, IN, NC, MA, and Vpr

29935	RPA4	replication protein A4, 34 kDa	Replication protein A and HIV-1 nucleocapsid protein interfere with the strand displacement DNA synthesis of HIV-1 reverse transcriptase by binding to the displaced strand and keeping it away from the newly synthesized strand

50810		hepatoma-derived growth factor, related protein 3	Hepatoma-derived growth factor 2 (HRP2) restores salt-stripped HIV-1 preintegration complex (PIC) activity in vitro

60489		apolipoprotein B mRNA editing enzyme, catalytic polypeptide-like 3G	Vif-negative HIV-1 produced from 293T cells transiently expressing hA3G are impaired in early and late viral DNA production, and in viral infectivity, which are correlated with an inability of tRNA(3)(Lys) to prime reverse transcription

A table of human proteins from the NIAID HIV-1 interaction database which are known to interact with HIV-1 RT and expressing the drug response predicting ELMs

**Table 3:** Biological Context

**Category**	**Term**	**Count**	**%**	**p-value**
GO BP Level 5	GO:0016310~phosphorylation	13	39.39%	5.21E-8

GO BP Level 5	GO:0008219~cell death	10	30.30%	7.86E-5

GO BP Level 5	GO:0006260~DNA replication	6	18.18%	9.30E-5

GO BP Level 5	GO:0006915~apoptosis	9	27.27%	3.16E-4

GO BP Level 5	GO:0006935~chemotaxis	5	15.15%	4.02E-4

GO MF Level 5	GO:0004697~protein kinase C activity	7	21.21%	1.22E-12

GO MF Level 5	GO:0004672~protein kinase activity	11	33.33%	1.75E-6

GO MF Level 5	GO:0032559~adenyl ribonucleotide binding	15	45.45%	1.97E-6

GO MF Level 5	GO:0003697~single-stranded DNA binding	5	15.15%	6.65E-6

GO MF Level 5	GO:0004707~MAP kinase activity	2	6.06%	0.0395

KEGG PATHWAY	hsa04650:Natural killer cell mediated cytotoxicity	10	30.30%	8.77E-9

KEGG PATHWAY	hsa04664:Fc epsilon RI signaling pathway	8	24.24%	1.30E-7

KEGG PATHWAY	hsa04530:Tight junction	9	27.27%	3.36E-7

KEGG PATHWAY	hsa04370:VEGF signaling pathway	7	21.21%	1.05E-6

KEGG PATHWAY	hsa04912:GnRH signaling pathway	6	18.18%	1.24E-4

KEGG PATHWAY	hsa05223:Non-small cell lung cancer	5	15.15%	1.34E-4

Gene Ontology categories (level 5) and KEGG pathways associated with the host proteins listed in Table 2. Count refers to the number of proteins from Table 2 which have the associated term. P-values were determined using the DAVID enrichment tool using the set of all human proteins with the ELMs in Table 2 as a background set.

## Discussion

The deadly course of HIV infection eventually leading to AIDS and associated opportunistic infections has been altered for a majority of individuals under HAART therapies thanks to combination antiretroviral therapies. These therapies have also reduced viral load dramatically in most patients, rendering them much less effective in transmitting the virus to others [53]. Research has focused on discovering new drugs targeting HIV proteins as well as on identifying host proteins necessary for viral growth as further possible targets for drugs. However, the interaction between the viral and host genotypes jointly affecting an individual's response to antiretroviral drugs has not been fully explored.

In this study we hypothesized that those host sequence motifs that are involved in protein-protein and protein/DNA/RNA interactions and also found in viral genomes are features that could play important roles in determining HIV-1 disease progression. Our prediction technique determines whether a particular therapy regimen is complementary to the sequence profile of each patient. Our thinking is motivated by the accumulating experimental evidence that viruses utilize motifs found in the host genome and proteins for integrating into host cell molecular networks and hijacking their function for viral replication [54,55]. Using linear sequence motifs shared by both the host and the virus provides an approach for investigating the plausible mechanisms of host virus interactions and suggesting those that may be altered by antiretroviral drugs.

We have used known resistance sites and host motifs found on HIV reverse transcriptase as features for differentiating responders from non-responders (or weak responders) in stepwise logistic regression for 16 different combinations of antiretroviral drug regimens containing at least one drug against HIV reverse transcriptase. Responder phenotype was defined multiple ways to gain insights into drug response at 8 weeks (SD phenotype classification) and 24 weeks (BM phenotype classification) after the beginning of the therapy and somewhere in between (IR Phenotype classification). Host motifs that appear to be highly relevant to viral replication such as the transcription site binding motifs [56,57] and miRNA binding site sequence motifs [58,59] could not be included into the analysis because these motifs are not contained within the RT region. Two resistance sites on HIV RT were found to be indicators of negative outcome, especially for regimens consisting of antiretrovirals targeting RT, but their influence was lower in HAART therapies. For the HAART therapy cases, the ELMS that contained these two resistance sites could be deleted from the model without sacrificing prediction accuracy. On the other hand, a number of ELMs were strongly correlated with positive outcome at different stages of antiretroviral therapy. These ELMs were associated with binding events leading to phosphorylation, ubiquination and the innate immune response.

Our approach to relate HIV sequence motifs to the course of infection does not require *a priori *information about how the HIV sequence would mutate in the presence of antiretroviral drugs. We were able to make accurate predictions without the resistance site information available in the literature. The input to our machine learning algorithm is simply the HIV sequence. We use publicly available bioinformatics tools to annotate these sequences with host motifs relevant to outcome. We then identify the motifs on the sequence that differentiate between responders and non-responders. These motifs can then be linked to specific viral host protein interactions and the pathways of these interactions. The promise of our approach will be fully explored with the availability of clinically annotated HIV whole genome sequences obtained at different time points during HAART therapy.

## Conclusion

Linear binding motifs found in both the host and viral proteomes constitute a set of features highly predictive of response to therapy involving different combinations of antiretroviral drugs. Stepwise logistic regression as used here utilizes only the HIV-1 sequence and does not require annotations of resistance sites specific to various antiretroviral drugs. This study emphasizes finding sequence motifs which facilitate binding between viral and host proteins. This binding may allow the hijacking of host protein binding sites from their usual binding partners and thus alter the signalling pathways of the host cell. Our study points to competitive binding of HIV proteins to host proteins using motifs found in the host as the mechanism of interplay between the host and pathogen genotypes in dictating response to therapy. Our method is applicable to other viral infections where the viral sequence is known but resistance sites to antiviral therapies have not yet been documented.

## Methods

### Data sources for HIV1 sequences and clinical phenotype assignment

This study utilizes sequence and clinical data from two distinct sources. All whole genome HIV-1 sequences were downloaded from the Los Alamos HIV Sequence Database  in order to get a motif expression map of the whole genome. As of 9/1/2006, this dataset consisted of 1,112 subtype B and 922 subtype C whole genome sequences, along with a smaller number of samples from other subtypes. This dataset also contained five reference sequences each for alignment of subtypes B and C.

We used data from the Stanford HIV Drug Resistance Database [33] in order to investigate the clinical relevance of host protein and DNA motifs on the RT region of the HIV-1 sequence. The Stanford database curates clinical information from drug trials on large HIV cohorts and associates them with the sequence coding the protein targeted by the drug. As of 11/15/2008, the database contained few PR region sequences. However, the dataset contained 2,019 RT sequences annotated with clinical parameters such as CD4 counts, VLs and the specific antiretroviral therapy as shown in Table 1. Each patient in this subset had at least 1 sequence fragment from RT, had 4 or more CD4 and VL measurements at 0, 2, 4, 8, 12, and 24 weeks during the course of a constant therapy regimen.

### Phenotype Classification

We focused on VL in the responder/non-responder classification [31] and examined the patient population using three methods of responder/non-responder classification: Standard Datenum (SD), Incremental Reduction (IR) and BiModal Classification (BM). The Standard Datenum method labels patients as responders if their VL decreases by 100-fold over 8 weeks of therapy [39]. The reduction in VL over the 24 week period logged by the Stanford HIV Drug Resistance Database exhibited a bimodal distribution for the patient population. Parameters of this distribution were obtained using the expectance maximization method described in [32] and indicated that a reduction of 2000 copies/mL in viral load would accurately split the responder and non-responder distributions. We refer to this method as bimodal classification. The third method we used was designed to avoid potential noise issues that could arise from relying the VL measurement on a single clinical visit [60]. The phenotype classification according to incremental reduction of the VL is such that if a patient's VL decreases between at least four visit pairs, then those patients are labelled as responders.

### Linear Motifs on HIV Genome and Proteome and Resistance Sites

Our classification method uses the presence and absence of short linear motifs on the HIV genome. These motifs can be grouped into three basic types: eukaryotic linear motifs (ELMs), nucleotide-based motifs and *a priori*-based resistance mutations. In order to evaluate the relative positions of nucleotide motifs and protein motifs on the same platform, we annotated the protein motifs back to their corresponding nucleotide positions. This could create some ambiguity since HIV has multiple overlapping reading frames. However, our clinical dataset only contained sequences from the RT region. We used a local BLASTx query [61] on a database of HIV-1 subtype B and C reference samples to translate the nucleotide fragments into their corresponding protein sequences (see Additional file 1). This ensured the proper translation even if the start and stop codons were missing from the sequence.

The first feature group consisted of ELM ligation sites and subcellular targeting sequences. These were identified on HIV-1 protein amino acid sequences using the ELM webserver tool [36]. The webserver tool filters out ELMs that fall into the globular regions proteins due to their predicted location within the 3D structure of the protein [36]. The second feature group consisted of HIV-1 sequence motifs that corresponded to annotated human transcription factor (TF) binding site motifs and miRNA binding sites. We used the MATCH™ web server [62] to annotate the TF binding sites on HIV-1 sequences with the public version of the TRANSFAC ^® ^database as of 11/14/08 [34]. We required a core similarity of 0.75 and a global similarity of 0.70 in parameter assignment and chose among alternatives the method that minimized false negatives [62]. For the annotation of miRNA binding sites, recognition sequences for human miRNA were obtained from a human miRNA database [35]. As of 11/14/08 this database contained 417 experimentally verified human miRNA binding recognition sequences. The HIV sequences were scanned using the RNAhybrid program [63] and the background parameters of the extreme value distribution were created from 1,000 random sequences with dinucleotide distributions identical to our compiled HIV-1 sequence database [63]. Any binding site which had a p < 0.01 was annotated as a potential miRNA binding site. The third group of features consisted of resistance mutation sites on HIV sequence [64]. In order to capture the known HIV-1 therapy resistance mutation sites on the amino acid sequence of RT, we created regular expressions similar to ELMs which identify the known resistance conferring mutations (RSs) from the Stanford HIV-1 Resistance Database [33].

### Predicting Therapy Outcome

We used stepwise logistic regression (SWLR) to assess the potential of the extracted short linear sequence features along the RT sequence in differentiating between responders and non responders [44]. SWLR was implemented in the MATLAB™ 2007b Statistics toolbox [65] (see Additional file 1). This regression method employs an iterative algorithm to determining the features that should be included in a predictive model. We used p-value < 0.01 as an entrance cutoff and p-value > 0.1 as a removal cutoff. In our study the algorithm converged to a final solution within 50–200 iterations.

SWLR algorithm was applied to differentiate responders from non-responders in three different assignments of the phenotype for 500 iterations of 2-fold cross validation. Since the efficiency of the SWLR algorithm is sensitive to the class composition of the training data [44] we ensured that each training set consisted of roughly 50% responders and 50% non-responders. After each set of training we determined the specificity and sensitivity of our classifier on the independent testing data and plotted the receiver operator characteristics (ROC) curve for each iteration in our scheme. The area under the ROC curve (AUC) represents the likelihood that one can identify a responder accurately using the method. This procedure was performed independently for each therapy regime under consideration and for the whole population shown in Table 1.

## Competing interests

The authors declare that they have no competing interests.

## Authors' contributions

WD wrote the manuscript with AT and performed the analysis with PE. LU provided technical insight. All authors approved the final manuscript.

## Pre-publication history

The pre-publication history for this paper can be accessed here:



## Supplementary Material

Additional file 1 **Source Code**. All python and MATLAB code required to produce the figures and tables shown in the manuscript. Documentation and unit-tests are provided to facilitate their usage. Click here for file
